# Parents' Knowledge of and Attitude Toward the Human Papillomavirus Vaccine in the Western Region of Saudi Arabia

**DOI:** 10.7759/cureus.32679

**Published:** 2022-12-19

**Authors:** Safa H Alkalash, Faisal A Alshamrani, Ethar H Alhashmi Alamer, Ghaida M Alrabi, Faisal A Almazariqi, Hadeel M Shaynawy

**Affiliations:** 1 Community Medicine and Health Care, College of Medicine, Umm Al-Qura University, Al Qunfudah, SAU; 2 Family Medicine, Menoufiya University, Shebin el Kom, EGY; 3 Medicine, College of Medicine, Umm Al-Qura University, Al Qunfudah, SAU; 4 Family Medicine, Urwah Primary Health Care, Madinah, SAU

**Keywords:** human papillomavirus (hpv), saudi parents, attitude, knowledge, hpv vaccine

## Abstract

Background: Human papillomavirus (HPV) is the most common cause of cervical cancer among Saudi females in reproductive age. Parents' awareness of and attitude toward vaccination against HPV in young females are very crucial to prevent the development of cervical cancer. This study aimed to assess the knowledge of and attitude toward the HPV vaccine among Saudi parents in the Saudi western region.

Methods: An analytical cross-sectional questionnaire-based study was conducted that included 343 parents randomly selected from in the Saudi western region. The online questionnaire was disseminated via WhatsApp and Telegram groups of parents in schools and among visitors of parents in primary health care.

Results: The studied parents had poor knowledge of HPV and its vaccine. About a third (32.9%) knew about the HPV vaccine and the most common source of their knowledge was physicians (38%) while the most frequent barrier for vaccination was their confidence of being not at risk (75.2%). About 90.0% of parents having a good level of knowledge were willing to vaccinate their children.

Conclusion: This study reveals a poor level of knowledge about HPV infection and its vaccine among both male and female parents in the Saudi Arabian western area. As a consequence, only 7.2% of them had vaccinated their female children. The majority of the parents having good knowledge about the HPV vaccine were willing to vaccinate their children. Therefore, this study highlights the necessity of educating women on cervical cancer risk factors, as well as the importance of screening programs.

## Introduction

Human papillomaviruses (HPVs) are small, non-enveloped, icosahedral, double-stranded deoxyribonucleic acid (DNA) viruses with a diameter of 52-55 nm [1]. HPV is considered to be the most prevalent sexually transmitted disease (STD), affecting the cutaneous and mucosal epithelia [2]. Most subtypes of HPV do not result in any lesions, warts, or symptoms. They are referred to as non-oncogenic, or low-risk types, and are associated with genital warts, while others are referred to as oncogenic, or high-risk types, that can result in cancers or intraepithelial neoplasia [2-4]. HPV can cause cancers of the oropharynx, vagina, anus, and penis [2]. In addition, HPV is responsible for about 99% of cervical cancer cases associated with types 16, 18, 31, 33, 35, 39, 45, 51, 52, 56, 58, 68, 73, 82, 26, 53, and 66 [4,5]. Cervical cancer is the fourth most common cancer in women, and also, the fourth leading cause of cancer death among women worldwide [6].

According to Global Cancer Observatory database, there were 604,000 new cases and 342,000 deaths from cervical cancer worldwide in 2020. Also, variations in morbidity and mortality rates were seen geographically [7]. Additionally, it was found to be correlated with national income as determined by the World Bank, ranging from 16.1 instances per 100,000 person-years in low-income nations to 9.2 in lower-middle-income countries to 6.9 in high-income and upper-middle-income countries [8-12]. Globally, there were 13.1 new cases of cervical cancer per 100,000 women (assessed age-standardized incidence rate, or ASIR) [10].

Cervical cancer is ranked as the 13th most common malignancy in women in Saudi Arabia. In women between the ages of 15 and 44, cervical cancer is ranked as the sixth most prevalent cancer with an incidence rate of 1.9 incidents per 1000,000 women [8]. Saudi Arabia is regarded as one of the countries with the lowest incidence of cervical cancer associated with a low prevalence of HPV. Every year, there are 152 new instances of cervical cancer, and there are 55 cases of fatality [3]. Recent estimates showed that 6.51 million Saudi Arabian women aged 15 years or older are at a high risk of developing cervical cancer, according to the World Health Organization [9]. In most parts of the world, cervical cancer incidence and mortality rates have decreased during the past few decades [7].

The HPV vaccine reduces the risk of cervical cancer by 70% through primary prevention of HPV types 16 and 18, which are the high-risk kinds [5]. There are still challenges, such as sociodemographic factors (religion, education level, age, gender, employment/unemployment, and the number of children), cultural norms, and socioeconomic factors (lower income and education level corresponding to a lower HPV vaccine uptake) [2]. Parents decide whether or not to vaccinate their children against HPV, and this decision-making process is influenced by a number of variables. Individual attitudes, knowledge, and health behaviors are among these determinants, as are people's concerns about the vaccine's long-term safety and its negative effects [6].

The Centers for Disease Control and Prevention (CDC), and the Food and Drug Administration (FDA) both recommended the HPV vaccine for both sexes in 2006. The HPV vaccine, which is typically given to females between the ages of 12 and 26 in three doses over the course of six months, has been effective as a primary preventative intervention against cervical cancer. Its use has been linked to a decrease in HPV-related illnesses, malignancies, and HPV genotype prevalence, with no documented negative effects [13,14]. The HPV vaccine has been accessible in the Kingdom of Saudi Arabia (KSA) for the past 10 years, and it is provided in major hospitals, with or without payment, to women with a doctor's prescription [14].

Parents' awareness about and attitude toward vaccines, like that for HPV, could affect their adherence to vaccinate their girls; hence, this study aimed to assess the knowledge and attitude of Saudi parents in the western region of Saudi Arabia toward the HPV vaccine.

## Materials and methods

Study design and setting

An analytical cross-sectional questionnaire-based study was carried out among Saudi parents over a six-month period from June to November 2022. A pre-designed questionnaire based on previous studies was used to collect information regarding awareness and acceptance of the HPV vaccine among parents [15,16]. The study was conducted in the western region, including Madinah, Makkah, Jeddah, Al Qunfudah, Taif, and Al Ardiyat, of Saudi Arabia. An ethical approval was provided by the Medical Research and Ethical Committee of the College of Medicine, Umm Al-Qura University, Makkah (UQU reference FEGL260822). The confidentiality of the anonymously collected data was maintained all the time.

Pilot study

A pilot study of 35 participants was undertaken to check language clarity and question understandability; the survey was slightly amended prior to use to reflect the outcomes of the pilot trial. All data from this pilot study acted as a guidance for the study researchers, and was excluded from the main study results. Finally, the test-retest technique was used to ensure questionnaire reliability.

Study sample and procedure for data collection

A sample size of 343 was calculated using EPI Info^TM ^(CDC, Atlanta, GA) based on the size of the total population in the western region, which was 8,325,304, at a 95% CI and a 5% margin of error. Data were collected from a convenient sample of 343 Saudi parents by using an online questionnaire; a link to the survey was distributed to respondents, via WhatsApp groups, and Telegram groups of parents in schools, and was also distributed to visitors of parents in primary health care (PHC). The questionnaire consisted of three sections. The first section included items to collect data about participants' demographics (age, gender, marital status, educational level, employment, monthly income), and to inquire about having a female child. The second section assessed their knowledge about HPV and HPV vaccine that included nine questions; the last question asked whether the participant needed education about the human papillomavirus. The third section was to assess their attitude toward HPV vaccine, and willingness to vaccinate their female children with the HPV vaccine, and barriers for refusing vaccination, in addition to a question about previous children's HPV vaccination, and whether they had an access to the content of the children's vaccination book.

The number of total collected questionnaires was 366. Incomplete questionnaires (n = 23) were discarded, so the total number of valid and complete questionnaires was 343.

Data analysis

Data were statistically analysed using the IBM SPSS Statistics, version 26 (IBM Corp., Armonk, NY). Participants who had at least 80% correct answers were considered as having good knowledge while below 80% was considered a poor level of knowledge. Data analysis was further subdivided into a descriptive phase, where categorical variables were described using frequencies and percentages, and an analytical phase, where the relationship between the variables was demonstrated by Fisher's exact test and chi-squared test (χ^2^), which were applied to qualitative data. A P-value less than 0.05 was regarded as statistically significant.

## Results

In our study, 39.1% of studied parents had an age ranging from 36 to 45 years; 66.2% were females and 86.3% were married (Table 1). Of them, 52.2% had university education and 67.9% were employed. Almost one third (33.8%) of the participants had a monthly income of 10,000-20,000 Saudi Riyals (SR) and 68.8% had a female child.

**Table 1:** Distribution of studied participants according to their demographics (N = 343) SR, Saudi Riyals All values are presented as numbers (N) and percentages (%).

Variable	N	Percentage
Age (years)		
15-25	48	14.0
26-35	76	22.2
36-45	134	39.1
>45	85	24.8
Gender		
Female	227	66.2
Male	116	33.8
Marital status		
Widow	12	3.5
Married	296	86.3
Divorced	35	10.2
Education level		
Intermediate school	58	16.9
Secondary school	18	5.2
Diploma	53	15.5
University	179	52.2
Postgraduate	35	10.2
Employment status		
Employed	233	67.9
Unemployed	97	26.3
Student	13	3.8
Monthly income (SR)		
<5000	48	14.0
5000-10,000	94	27.4
10,000-20,000	116	33.8
>20,000	26	7.6
None	59	17.2
Has a female child		
No	107	31.2
Yes	236	68.8

A total of 34.7% parents had previous knowledge about HPV, 37.9% thought that HPV is very dangerous, and only 32.7% knew the harmful association between HPV infection and occurrence of cervix cancer (Table 2). The most common source of their knowledge was physicians (38.0%). A total of 19.2% thought that the vaccine has side effects, and 35.3% reported that it prevents HPV infection. The majority (87.8%) reported the need for education about HPV.

**Table 2:** Distribution of studied participants according to their response to knowledge items regarding HPV and HPV vaccine (N = 343) HPV, human papillomavirus All values are presented as numbers (N) and percentages (%).

Variable	N	Percentage
Has knowledge about HPV		
No	224	65.3
Yes	119	34.7
HPV is very dangerous		
No	32	9.3
Yes	130	37.9
Maybe	181	52.8
Harm that is associated with the infection with HPV		
Cancer of the breast	1	0.3
Cancer of the anus, penis, vagina, vulva, and the back of the throat	1	0.3
Cancer of the cervix	112	32.7
Negatively affects fertility	25	7.3
Unknown	204	59.5
Has knowledge about the HPV vaccine		
No	230	67.1
Yes	113	32.9
Source of knowledge about the HPV vaccine (N = 113)		
Physicians	43	38.0
Internet	33	29.2
Family and friends	8	7.0
Social media	29	25.8
Vaccine has any side effects		
No	73	21.3
Yes	66	19.2
Maybe	204	59.5
Vaccine prevents the infection with HPV		
No	27	7.9
Yes	121	35.3
Maybe	195	56.9
Vaccine reduces the severity of symptoms after getting infected		
No	33	9.6
Yes	165	48.1
Maybe	145	42.3
Target group for the vaccine		
Unmarried females 9 to 25 years old	98	28.6
Married females 9 to 25 years old	37	10.8
Before marriage	22	6.4
Don't know	186	54.2
Facility that can introduce the HPV vaccine		
Schools	2	0.6
Primary health care	133	38.8
Hospitals	42	12.2
Don't know	166	48.4
Need for more education about HPV		
No	42	12.1
Yes	301	87.8

A total of 58.6% of studied parents were willing to vaccinate their children with the HPV vaccine (Table 3). For those who refused vaccination, the most common barrier was their thought of not being at risk of infection (75.2%). More than half of parents (59.5%) had a conviction about the effectiveness of the vaccine for their children, but only 7.2% had a child vaccinated against HPV.

**Table 3:** Distribution of studied participants according to their attitude toward and willingness to vaccinate their children with the HPV vaccine (N = 343) HPV, human papillomavirus All values are presented as numbers (N) and percentages (%).

Variable	N	Percentage
Willingness to vaccinate their children		
No	52	15.2
Yes	201	58.6
Maybe	90	26.2
Reason for the refusal to vaccinate their children (N = 52)		
Vaccine is expensive	0	0.0
Afraid of the side effect of the vaccine	1	1.9
Don't believe in the effectiveness of the vaccine	4	7.6
Don't have a family history for cervical cancer	1	1.9
Don't have enough knowledge about the vaccine	7	13.4
Not at risk to be affected	39	75.2
Conviction about the effectiveness of the vaccine for their children		
No	45	13.1
Yes	204	59.5
Maybe	94	27.4
Have any of their children received the HPV vaccine (for those having a female child) (N = 236)		
No	219	92.8
Yes	17	7.2
Have access to the content of their children's vaccination book		
No	80	23.3
Yes	263	76.7

**Figure 1:**
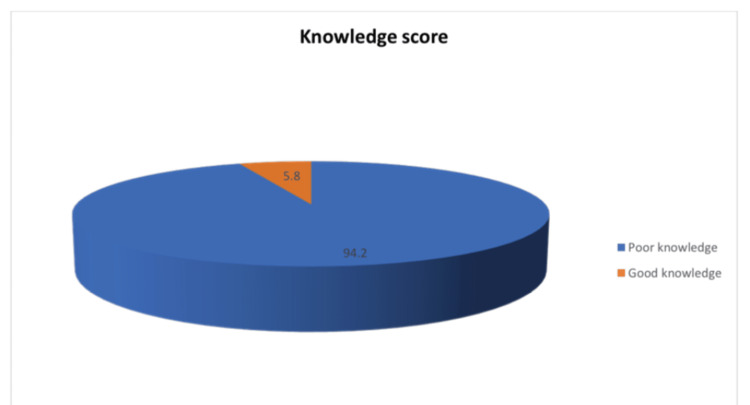
Percentage distribution of participants according to their knowledge level regarding HPV and HPV vaccine HPV, human papillomavirus

As shown in Figure 1, 94.2% of parents had a poor knowledge level regarding HPV and HPV vaccine, while 5.8% had a good knowledge level.

Employed parents, those who got their knowledge from physicians, who had access to the content of the children's vaccination book, and who had a conviction about the effectiveness of the vaccine for their children formed a significantly high percentage of those having a good knowledge level (P = 0.028, <0.001, 0.003, 0.011, 0.004; Table 4).

**Table 4:** Relationship between the knowledge level and participants' demographics (N = 343) HPV, human papillomavirus; SR, Saudi Riyals All values presented as numbers (N) and percentages (%). *Statistically significant (P < 0.05).

Variable	Knowledge level				P-value
	Poor		Good		
	N	Percentage	N	Percentage	
Age (years)					0.269
15-25	46	14.2	2	10.0	
26-35	73	22.6	3	15.0	
36-45	122	37.8	12	60.0	
>45	82	25.4	3	15.0	
Gender					0.115
Female	217	67.2	10	50.0	
Male	106	32.8	10	50.0	
Marital status					0.542
Widow	12	3.7	0	0.0	
Married	279	86.4	17	85.0	
Divorced	32	9.9	3	15.0	
Education level					0.124
Intermediate school	18	5.6	0	0.0	
Secondary school	58	18.0	0	0.0	
Diploma	49	15.2	4	20.0	
University	167	51.7	12	60.0	
Postgraduate	31	9.6	4	20.0	
Employment status					0.028*
Employed	214	66.3	19	95.0	
Unemployed	96	29.7	1	5.0	
Student	13	4.0	0	0.0	
Monthly income (SR)					0.166
<5000	47	14.6	1	5.0	
5000-10,000	89	27.6	5	25.0	
10,000-20,000	108	33.4	8	40.0	
>20,000	22	6.8	4	20.0	
None	57	17.6	2	10.0	
Have female children					0.107
No	104	32.2	3	15.0	
Yes	219	67.8	17	85.0	
Source of knowledge about the HPV vaccine					<0.001*
Physicians	30	9.3	13	65.0	
Internet	29	9.0	4	20.0	
Family and friends	8	2.5	0	0.0	
Social media	26	8.0	3	15.0	
Have any of their children received the HPV vaccine (for those having a female child) (N = 236)					0.003*
No	206	63.8	13	65.0	
Yes	13	4.0	4	20.0	
Have access to the content of their children's vaccination book					0.011*
No	80	24.8	0	0.0	
Yes	243	75.2	20	100.0	
Have a conviction about the effectiveness of the vaccine for their children					0.004*
No	45	13.9	0	0.0	
Yes	185	57.3	19	95.0	
Maybe	93	28.8	1	0.5	

Married parents, who had university education, and who got their knowledge from physicians formed a significantly high percentage of those having a positive attitude and willing to vaccinate their children with the HPV vaccine (P = 0.011, 0.043, 0.001; Table 5).

**Table 5:** Relationship between participants' willing to vaccinate their children and their demographics (N = 343) HPV, human papillomavirus; SR, Saudi Riyals All values are presented as numbers (N) and percentages (%). *Statistically significant (P < 0.05).

Variable	Willing to vaccinate their children							P-value
	Maybe			No		Yes		
	N		Percentage	N	Percentage	N	Percentage	
Age (years)								0.079
15-25	10	11.1		6	11.5	32	15.9	
26-35	15	16.7		11	21.2	50	24.9	
36-45	43	47.8		15	28.8	76	37.8	
>45	22	24.4		20	38.5	43	21.4	
Gender								0.818
Female	62	68.9		34	65.4	131	65.2	
Male	28	31.1		18	34.6	70	34.8	
Marital status								0.011*
Widow	1	1.1		5	9.6	6	3.0	
Married	74	82.2		42	80.8	180	89.6	
Divorced	15	16.7		5	9.6	15	7.5	
Education level								0.043*
Intermediate school	4	4.4		5	9.6	9	4.5	
Secondary school	20	22.2		12	23.1	26	12.9	
Diploma	10	11.1		2	3.8	41	20.4	
University	48	53.3		28	53.8	103	51.2	
Postgraduate	8	8.9		5	9.6	22	10.9	
Employment status								0.709
Employed	57	63.3		34	65.4	142	70.6	
Unemployed	30	33.3		16	30.8	51	25.4	
Student	3	3.3		2	3.8	8	4.0	
Monthly income (SR)								0.904
<5000	11	12.2		8	15.4	29	14.4	
5000-10,000	22	24.4		13	25.0	59	29.4	
10,000-20,000	34	37.8		20	38.5	62	30.8	
>20,000	7	7.8		2	3.8	17	8.5	
None	16	17.8		9	17.3	34	16.9	
Has a female child								0.189
No	25	27.8		12	23.1	70	34.8	
Yes	65	72.2		40	76.9	131	65.2	
Source of knowledge about the HPV vaccine								0.001*
Physicians	5	5.6		5	9.6	33	16.4	
Internet	1	1.1		4	7.7	28	13.9	
Family and friends	3	3.3		1	1.9	4	2.0	
Social media	6	6.7		2	3.8	21	10.4	
Have any of their children received the HPV vaccine (for those having a female child) (N = 236)								0.057
No	64	71.1		38	73.1	117	58.2	
Yes	1	1.1		2	3.8	14	7.0	
Have access to the content of their children's vaccination book								0.952
No	20	22.2		12	23.1	48	23.9	
Yes	70	77.8		40	76.9	153	76.1	

**Figure 2:**
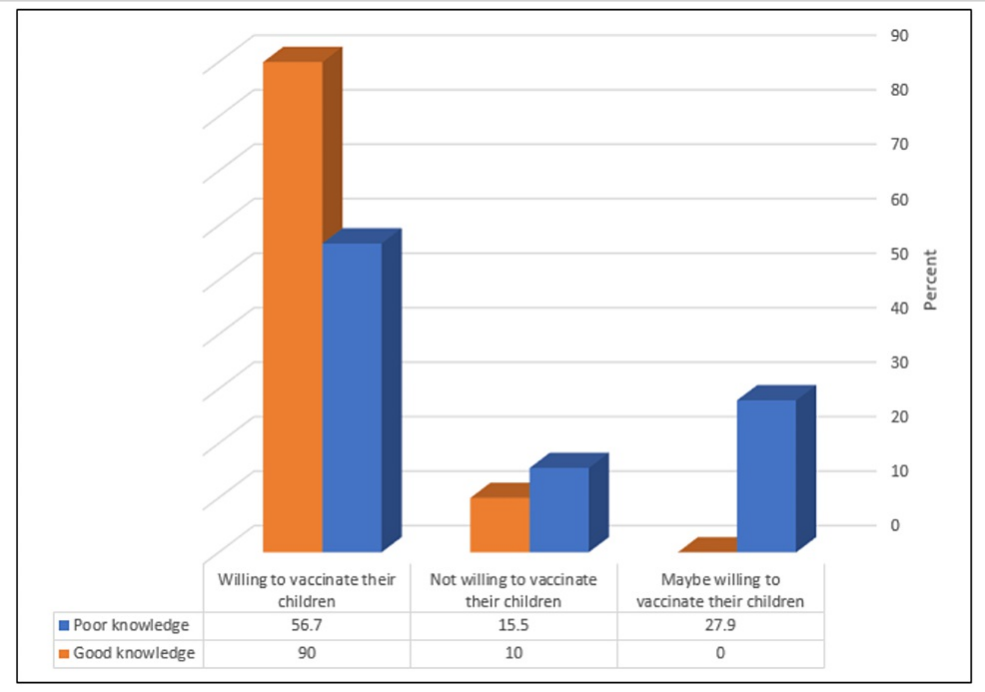
Relationship between the knowledge level and participants' attitude and willingness to vaccinate their children with the HPV vaccine (N = 343) Fisher's exact test = 8.07, P = 0.021

As shown in Figure 2, parents who had a positive attitude and were willing to vaccinate their children with HPV formed a significantly high percentage of those with a good knowledge level (P = 0.021).

## Discussion

It has become critical to promote cervical cancer awareness in the Kingdom of Saudi Arabia because knowledge and awareness are important components when adopting healthy behaviors and accepting newly proposed preventative methods. According to the World Health Organization, HPV accounts for 99% of cervical cancer cases [17]. The HPV vaccine against the strains linked with cervical cancer has been on the market since 2006; nonetheless, much of the current data paints a picture of pervasive misunderstanding about HPV infection and vaccination among the target population [18]. Although there is a rising interest in community health awareness programs in Saudi Arabia, the majority of current researches focus on breast cancer, diabetes, and obesity. In contrast, only few studies with small sample sizes have been undertaken to examine Saudis' knowledge about cervical cancer, and uptake of HPV vaccination [19].

Participants in the current study, which covered the Saudi western region, had poor knowledge of HPV. The study found that 34.7% of parents had previous knowledge about HPV, 37.9% thought that HPV is very dangerous, and only 32.7% knew that the harm associated with HPV infection is cancer of the cervix. The cause for this poor level of knowledge regarding HPV and its vaccine may be that in KSA, the population usually knows only about the common compulsory vaccinations for their children, but not a new and unusual vaccine like HPV. Another cause for this poor knowledge, from our point of view, may be the insufficient health education from the side of primary health care. Therefore, it is recommended to increase public awareness about the HPV vaccine through encouraging their healthcare providers in primary care settings.

These findings are similar to a survey of 181 Saudi medical students done at King Faisal University in 2014, where the majority of the students had little understanding of early warning signs, symptoms, and risk factors for cervical cancer. The percentages of accurate answers varied from 43.7% to 55% on average [20]. Furthermore, the overall knowledge of cervical cancer among students aged 17 to 26 in Poland was found to be insufficient, and HPV infection was not thought to be the primary cause [21]. Surprisingly, a recent survey among healthcare practitioners in Greece revealed a considerable knowledge gap with HPV, with just 30% of the sample appearing to be aware of HPV playing a big part in carcinogenesis. According to the findings of a recent Thai poll, the level of understanding regarding HPV was quite low [22].

About a third of the current study participants (32.9%) had knowledge about the HPV vaccine; 19.2% of them thought that the vaccine had different side effects and the most common source of their knowledge was their physicians (38.0%). These results are the same as those obtained by a previous study in Saudi Arabia done in 2014, where 67% of the participants were unaware of the existence of HPV vaccination [20].

The majority of the current study subjects (87.8%) reported their need for health education about HPV. This is a very amenable issue because it reflects the readiness of public to improve their awareness level. Therefore, imparting community education is a mandatory action.

Moreover, this study found that 58.6% of parents were willing to vaccinate their children with the HPV vaccine, while 26.2% reported that they "may" vaccinate them. For those who refused vaccination, the most common barrier was their thought of not being at risk of infection (75.2%). More than half of parents (59.5%) had a conviction about the effectiveness of the vaccine for their children, but only 7.2% had a vaccinated child against HPV. Of them, 76.7% had access to the content of the children's vaccination book. These findings are congruent with those of a previous research done in Riyadh in 2016, in which only 37% of participants declined the immunization [23]. Furthermore, in a recent national research study conducted in Saudi Arabia, around 55% were eager to take the HPV vaccination if provided, and 73% said they would recommend the HPV vaccine to others. Similarly, over 50% of Jeddah medical students showed desire in obtaining the HPV vaccination [24,25].

Good knowledge about HPV vaccination was detected among employed parents, those who received health-related information from their physicians, who had already vaccinated their female children, who had access to the content of their children's vaccination book, and those who believed in the effectiveness of the vaccine for their children. This outcome was not unexpected as employed parents might hear about the new trend of the HPV vaccine from fellow workers, specifically after it becoming an obligatory vaccine requested by the intermediate schools to be given to all female students. In addition, parents who sought medical information from physicians had more accurate knowledge about the HPV vaccine than peers who received this information from a non-specialized source. Careful parents, who were keen to know about their children vaccination status through their vaccination books, were able to know more about the HPV vaccine that was added to the new schedule. Through explaining the indication and health benefits of any new drug or vaccine, it is easy to convince someone to get it. Therefore, parents who had a good level of knowledge about the vaccination became more convinced about its effectiveness.

It was noticed that being married, highly educated and receiving health information from the physicians were all determinants for willingness of the parents to vaccinate their children. This finding is a logical one as married, highly educated parents could know more and easily understand the health benefits of this vaccine, a vaccine that would protect their children from a highly aggressive disease such as cancer of the cervix. As a result, they would accept this vaccine more easily than others. Physicians are also able to persuade their patients, and clients, to receive the vaccine by explaining its health benefits and using evidence-based medicine.

At the end of this study, 90.0% of the parents, who had a good level of knowledge about the HPV vaccine, were found willing to vaccinate their children (P = 0.021). This result ensures the great necessity to conduct more health educational campaigns all over the Kingdom of Saudi Arabia, and not only in the western region, in order to raise public awareness about this highly important issue. Protection of women from cervical cancer starts by educating the whole population about it, and spreading awareness about its primary prevention, vaccination against HPV, which is already available within all regional PHC facilities.

This study also had some limitations. First, the obtained data about the number of vaccinated children was self-reported; thus, there is a possibility of measurement bias as self-reporting depends on memorizing capabilities of the participants and their education levels. Second, the online approach for data collection may threaten the credibility of the obtained data. Third, study results were compared with the statistics on HPV from few countries other than Saudi Arabia while it was better to compare the awareness level of the Saudi population with that from many other different countries in order to explore whether lack of awareness is significant or it is similar to trends elsewhere. Despite these limitations, this study had many strength points that have to be acknowledged. The most important one is that it highlighted the acceptability level of Saudi parents about the HPV vaccine and put a spotlight on their poor level of awareness and practice regarding such an important vaccine. The next benefit was that the study was conducted on a large scale in many different, big cities such as Jeddah, Makkah, Madinah, Taif, and Al Ardiyat in addition to Al Qunfudah, which is a remote area belonging to the Saudi western region. Opening the route for many other researches to be conducted in this area is one of the advantages of this research. Finally, attracting the attention of health institutions to provide community education courses on cervical cancer and how to avoid it through early detection and vaccination could be an advantage of this study.

## Conclusions

This study reveals the poor level of knowledge about HPV infection and its vaccine among both male and female Saudi parents in the Saudi Arabian western area; even highly educated people claimed a lack of information. Health officials have recently begun promising nationwide HPV awareness initiatives to improve public understanding of HPV and the significance of vaccination. Therefore, through this study, we highlight the necessity of educating women about cervical cancer risk factors, as well as the importance of screening programs. Primary healthcare providers should provide knowledge about the HPV vaccine to their patients and clients, and encourage them to vaccinate their girls. A well-designed health education campaign regarding cervical cancer and the benefits of screening and immunization would increase public knowledge in Saudi Arabia.
